# Comparing Adverse Health Outcomes in Infants Born to Women Monoinfected with Zika Virus or Infected with Both Zika and Chikungunya Viruses during Pregnancy: A Prospective Cohort Study in Pernambuco, Brazil (2015–2017)

**DOI:** 10.4269/ajtmh.25-0512

**Published:** 2026-06-09

**Authors:** Gabriela Renata Neves Fulco, Ricardo Arraes de Alencar Ximenes, Ulisses Ramos Montarroyos, Celina Maria Turchi Martelli, Thalia Velho Barreto de Araújo, Elizabeth Brickley, Mariana de Carvalho Leal Gouveia, Liana Maria Viera de Oliveira Ventura, Maria Valquíria de Medeiros Silva, Juliana Menezes Soares de Souza Azevedo Fontes, Demócrito de Barros Miranda-Filho

**Affiliations:** ^1^ Programa de Pós-graduação em Ciências da Saúde, Universidade de Pernambuco, Recife, Brazil;; ^2^ Faculdade de Ciências Médicas, Universidade de Pernambuco, Recife, Brazil;; ^3^ Departamento de Medicina Tropical, Universidade Federal de Pernambuco, Recife, Brazil;; ^4^ Instituto de Ciências Biológicas, Universidade de Pernambuco, Recife, Brazil;; ^5^ Departamento de Saúde Coletiva, Instituto Aggeu Magalhães, Recife, Brazil;; ^6^ Departamento de Medicina Social, Universidade Federal de Pernambuco, Recife, Brazil;; ^7^ Department of Infectious Disease Epidemiology, London School of Hygiene & Tropical Medicine, London, United Kingdom;; ^8^ Departamento de Cirurgia, Universidade Federal de Pernambuco, Recife, Brazil;; ^9^ Departamento de Oftalmologia, Fundação Altino Ventura, Recife, Brazil

## Abstract

The association between Zika virus (ZIKV) infection in pregnant women and congenital defects is well-established. However, the consequences of infection with both ZIKV and Chikungunya virus (CHIKV) during pregnancy remain poorly understood. The aim for the present study was to estimate and compare the risk of adverse outcomes in infants born to mothers infected with ZIKV alone and those born to mothers infected with both ZIKV and CHIKV during pregnancy. A cohort study was conducted, following pregnant women with rash who were reported to the Strategic Health Surveillance Information Center of the State of Pernambuco between 2015 and 2017 and underwent laboratory testing for ZIKV and CHIKV. All participants resided within a 120-kilometer radius of the city of Recife, Pernambuco, Brazil. Newborns were monitored through standardized assessments conducted by specialists from the Microcephaly Epidemic Research Group. Adverse outcomes were compared between newborns prenatally exposed to ZIKV alone and those exposed to both ZIKV and CHIKV using descriptive statistics, as well as RRs. A total of 278 children (*n* = 198 exposed to ZIKV alone; *n* = 80 exposed to ZIKV and CHIKV) were included. No significant differences were observed between the groups in the offspring’s risks of microcephaly, low birth weight, prematurity, small for gestational age, dysphagia, neurological, ophthalmological, audiological, or neuroimaging abnormalities. Although previous studies have revealed that each infection independently may be associated with adverse outcomes in newborns, the present study generally revealed no difference in the frequency or type of adverse effects in children born to mothers infected with both ZIKV and CHIKV compared with those exposed only to ZIKV during pregnancy.

## INTRODUCTION

Beginning in early 2015, reports began to emerge of a novel exanthematic disease with a similar clinical presentation to dengue fever, which was later identified to be Zika virus (ZIKV). In August 2015, an increased frequency of cases of newborns with microcephaly was registered in the Brazilian State of Pernambuco. By November 2015, the Brazilian Ministry of Health announced an evidence-based association between ZIKV infection during pregnancy and the microcephaly epidemic.^1^

Various neurological abnormalities, including congenital microcephaly, have been observed among children prenatally exposed to ZIKV.^2^ Microcephaly is a clinical indicator that directly reflects neonatal brain development, although it can also be observed in healthy newborns.^3^ The association between microcephaly and ZIKV is already well-established, and the consequences for affected children include diminished quality of life and reduced life expectancy, with consequent impact on the lives of other family members.^4^ Notably, ZIKV does not cause microcephaly in all congenital infections, and other structural alterations and functional impairments may arise, compromising the acquisition of neuropsychomotor skills (cognition, language, behavior, and motricity), which can be visualized in brain scans or detected during subsequent neurodevelopmental evaluations.^5^ The range of clinical manifestations associated with ZIKV infection during pregnancy is collectively known as congenital Zika syndrome (CZS).^6^

Chikungunya virus (CHIKV), an arthropod-borne virus (arbovirus) of the genus *Alphavirus* and the family *Togaviridae*, can be transmitted by mosquitoes of the *Aedes* genus, particularly *Aedes aegypti,* which is the primary vector of ZIKV. Like ZIKV, CHIKV can be vertically transmitted from mother to child, and the period during gestation in which a woman is infected affects the severity of the disease in the newborn.^7^ When infection is transplacental, the virus passes directly into the bloodstream of the fetus, without taking the normal route through the epidermis, into the lymphatic system, and through the circulatory system to infect organs, where CHIKV replication occurs.^7^ In cases of intrapartum maternal viremia, CHIKV transmission can result in severe infection of the newborn, with symptomatic newborns typically developing clinical signs of Chikungunya disease between 3 and 7 days after birth. These may include fever, arthralgia, poor appetite, irritability, skin rashes, meningoencephalitis, and delays in the development of adaptive neurological functions.^8^ Some more serious manifestations of Chikungunya, including transient tachypnoea of the newborn, respiratory stress syndrome, sepsis, cyanosis, apnea, lethargy, abdominal distension, anuria, jaundice, hyposaturation, bradycardia, cardiac and kidney anomalies, pulmonary hemorrhage, and seizures, may lead to neonatal death.^9^

According to the Brazilian Ministry of Health’s Epidemiological Bulletin, 2016 and 2017 were the years with the highest incidence of Chikungunya cases, mostly in the Northeast Region. The Zika epidemic occurred during this same period and geographic region.^9^^,^^10^ Because both CHIKV and ZIKV are transmitted by the same vector, cocirculation of the viruses is frequent, and there is a high probability of coinfection in regions where *Aedes* spp. mosquitos are present. It should also be noted that the symptoms and clinical profile of the disease do not appear to differ significantly in cases of ZIKV monoinfection and coinfection with other arboviruses.^11^

In cases of coinfection during pregnancy, evidence remains limited, and it is not known whether the viruses can modify each other’s effects and influence the health outcomes for prenatally exposed offspring. One of the main questions that requires clarification is whether infection with two or more viruses may lead to more severe symptoms and consequences than monoinfection.^12^ The authors of previous studies have compared maternal symptoms between ZIKV monoinfection and sequential ZIKV/CHIKV infections during pregnancy^10^ and quantified the absolute and RRs associated with fetal exposure to ZIKV.^13^ However, there is insufficient evidence in the literature to determine whether coinfection with the CHIKV can lead to more severe consequences for the health of the newborn. Using data collected from maternal–child dyads in Northeast Brazil, the aim for the present study is to compare the risks of adverse outcomes in infants born to mothers infected with ZIKV alone and those born to mothers infected with both ZIKV and CHIKV during pregnancy.

## MATERIALS AND METHODS

A prospective observational cohort study was conducted between December 2015 and June 2017, including 278 women with a laboratory-confirmed diagnosis of ZIKV monoinfection during pregnancy and 80 women with a laboratory-confirmed diagnosis of both ZIKV and CHIKV infections during pregnancy.

Recruitment of participants to the Microcephaly Epidemic Research Group (MERG) Pregnant Women Cohort was initiated as soon as the State of Pernambuco received notification of a pregnant woman presenting with a skin rash. Women residing within a 120-km radius of the Recife Metropolitan Region in Pernambuco, Brazil, were included in the present study. Once the Centro de Informações Estratégicas de Vigilância em Saúde de Pernambuco was notified, blood samples were collected within 5 days of the appearance of the rash. After obtaining informed consent, MERG field researchers used questionnaires to gather data on women’s sociodemographic factors, reproductive history, clinical and behavioral aspects of the current pregnancy, vaccination history, and characteristics of the exanthem and the infection. To enhance diagnostic sensitivity for ZIKV and CHIKV infection during pregnancy, a combination of sequenced molecular and serologic tests was used. After ∼14 days, the MERG field researchers collected a second blood sample. After childbirth, a third blood sample was collected from women with live births. The occasional loss of a blood sample at one of the three time points did not prevent sample collection at a later time point.

Zika virus infection was confirmed using molecular (quantitative reverse transcription polymerase chain reaction [qRT-PCR]) and serological (IgM, IgG3, and the plaque reduction neutralization test [PRNT50]) assays. Chikungunya virus infection was determined using qRT-PCR and IgM testing. The principal exposure was maternal infection with both ZIKV and CHIKV during pregnancy, and this group was compared with pregnant women infected only by ZIKV. In cases of ZIKV monoinfection, the degree of laboratory evidence was classified according to the criteria adopted by Ximenes et al. (2019) as follows: robust evidence was defined as a positive qRT-PCR test result, seroconversion, or at least two positive serological test results during pregnancy, or testing positive for IgM or IgG3 during pregnancy in combination with a negative PRNT50 test result within 6 months after pregnancy; moderate evidence was defined as the patient testing positive for IgM or IgG3 during pregnancy, PRNT50 evidence of seroconversion, or an inconclusive PRNT50 test result during pregnancy followed by a positive PRNT50 test result within 3 months after childbirth; limited evidence of ZIKV infection was defined as a positive PRNT50 test result during pregnancy or in the 6 months after childbirth or evidence of seroconversion in the PRNT50 test conducted in the first 2 to 3 months after childbirth.^14^

Maternal ZIKV and CHIKV infection was established on the basis of an interpretation of molecular and serological assays for ZIKV and CHIKV by a panel of MERG specialists. All women were already classified for ZIKV status according to the criteria outlined by Ximenes et al. (2019), and CHIKV infection was classified into two categories. Robust evidence of CHIKV was defined as at least one positive qRT-PCR test result or seroconversion of IgM from negative to positive during pregnancy, whereas moderate evidence was defined as testing positive for CHIKV by IgM at least once after the seventh week of gestation. To define a case of ZIKV infection during pregnancy, the three levels of evidence were combined (robust, moderate, and limited), and for CHIKV infection, the two levels of evidence were combined (robust and moderate). Cases in which it was not possible to ascertain whether infection had occurred during pregnancy were excluded from the study. Infants’ health outcomes were evaluated during physical examinations by the MERG team of clinical specialists (pediatrician, neurologist, phono-audiologist, and ophthalmologist), with data recorded on standardized forms. The follow-up differed between the outcomes. The median age across the assessments ranged from 18.4 to 26.2 months, with the last assessment at 37.9 months of age. The raters were blinded to maternal infection status.

The independent variables chosen for the study included maternal sociodemographic data (age, race/skin color, years of schooling, and social class), reproductive history, fetal malformations in previous pregnancies, smoking, recreational drug use, type of delivery, diagnosis of ZIKV infection, and diagnosis of both ZIKV and CHIKV infections during pregnancy. The dependent variables included microcephaly, neurological, ophthalmological, and audiological abnormalities, dysphagia, and central nervous system (CNS) imaging anomalies. The purchasing power of individuals and families was defined by the use of the Brazilian economic classification criteria of 2015, which defines eight socioeconomic classes from A (highest) to E (lowest).^13^^,^^15^

Microcephaly was defined as a head circumference ≥2 SDs below the mean for age and sex, based on the WHO growth curves for infants born at term and on the INTERGROWTH-21st curves for infants born at <37 weeks of gestational age.^12^ To compare this outcome between the two groups, three different cutoff points (2, 2.5, and 3 SDs below the mean) were used for microcephaly. Neurological abnormalities included seizures, altered consciousness or behavior, localized motor deficits, altered tonus or trophism (atrophy or hypotrophy), and pyramidal signs. Ophthalmological abnormalities were detected using visual acuity testing, ocular motility testing, cycloplegic refraction, indirect ophthalmoscopy, and wide-angle fundus imaging (Retcam Shuttle, Clarity Medical Systems, Pleasanton, CA, USA). Audiological abnormalities included an abnormal response to the first screening, with a short-latency auditory brainstem response to stimuli. The definition of dysphagia was based on information from the mother or caregiver. Neuroimaging results from the transfontanelle ultrasound were assessed for calcifications, cerebellar hypoplasia or atrophy, agenesis or dysgenesis of the corpus callosum, and diffuse cortical atrophy.

For comparing the clinical features of ZIKV infection, the χ^2^ or Fisher’s exact tests were used for categorical variables, and Student’s *t*-test or the Mann-Whitney *U* test were used for continuous variables. In the bivariate analysis to compare the risks of adverse outcomes in infants born to mothers infected with ZIKV alone and those born to mothers infected with both ZIKV and CHIKV during pregnancy, for each of the adverse outcomes studied, the RR was estimated with a 95% CI. For outcomes with a frequency of 0, in some results, the odds ratio (OR) was calculated using the median-unbiased estimator for binary data in an exact logistic regression model.^1^^,^^2^ The significance level adopted was 5% (*P* <0.05), and data were analyzed using Stata version 14 (StataCorp LLC, College Station, TX, USA).

## RESULTS

The present study included a total of 278 maternal–child dyads who had tested positive for ZIKV alone (*n* = 198) or for both ZIKV and CHIKV during gestation (*n* = 80). The median maternal age of 26 years (interquartile range: 21 to 31 years) was the same for both groups.

Comparison of the women exposed to ZIKV with those exposed to both ZIKV and CHIKV revealed that there was no difference between the groups, except for the trimester of rash. Most cases of maternal rash were detected in the third trimester in both groups, although the frequency of infection in the third trimester was significantly higher in the monoinfected group. Most of the women in both groups did not smoke or use drugs recreationally during pregnancy (Table 1). According to the interpretation of molecular and serological assays, 55 of the pregnant women had moderate evidence of maternal CHIKV infection, whereas 25 had robust evidence.

**Table 1:** Characteristics of women monoinfected with Zika virus and women infected with both Zika virus and chikungunya virus during gestation in the Microcephaly Epidemic Research Group Pregnant Women Cohort

Variables	ZIKV (*n* = 198)	ZIKV + CHIKV (*n* = 80)	*P*-Value (χ^2^)
Age, years, median (IQR)	26 (21–31)	26 (21–31)	
Sociodemographic data, *n* (%)			
Race/skin			
“Preto” (i.e., black)	48 (24.24)	12 (15.00)	
“Pardo” (i.e., mixed race)	19 (9.60)	5 (6.25)	
“Branco” (i.e., white)	129 (65.15)	63 (78.75)	
Other	2 (1.01)	–	0.17 (Fisher)
Years of schooling			
0–9	82 (41.41)	28 (35.00)	
10–12	95 (47.98)	45 (56.25)	
13+	21 (10.61)	7 (8.75)	0.46 (Pearson)
Social class			
A, B1, and B2	18 (9.09)	7 (8.75)	
C1	30 (15.15)	10 (12.50)	
C2	74 (37.37)	36 (45.00)	
D and E	76 (38.38)	27 (33.75)	0.69 (Pearson)
Reproductive history			
Previous pregnancy			
Yes	132 (66.67)	57 (71.25)	
No	66 (33.33)	23 (28.75)	0.46 (Pearson)
Fetal malformation in previous pregnancies			
Yes	3 (2.63)	1 (1.85)	
No	111 (97.37)	53 (98.15)	
N/A	66	23	1.00 (Fisher)
Current pregnancy characteristics			
Smoking			
Yes	12 (6.06)	5 (6.25)	
No	186 (93.94)	75 (93.75)	1.00 (Fisher)
Recreational drug use			
Yes	2 (1.01)	1 (1.25)	
No	10 (5.05)	4 (5.0)	1.00 (Fisher)
Diagnosis of ZIKV/CHIKV during pregnancy			
Robust evidence	111 (56.1)	51 (63.7)	
Moderate evidence	20 (10.1)	8 (10.0)	
Limited evidence	67 (33.8)	21(26.3)	0.45 (Pearson)
Trimester of rash*			
First	24 (12.44)	21 (26.58)	
Second	65 (33.68)	27 (34.18)	
Third	104 (53.89)	31 (39.24)	0.01 (Pearson)

CHIKV = chikungunya virus; IQR = interquartile range; ZIKV = Zika virus.

The numbers are varied because of missing values.

* In the group of ZIKV-monoinfected women, there were three cases without a rash; in the group infected with both ZIKV and CHIKV, there was one case without a rash and two cases with no information about a rash.

A comparison of the RR for women with ZIKV monoinfection versus those infected with both ZIKV and CHIKV, in relation to the severity of microcephaly, is shown in Table 2 using three cutoff points (–2 SDs, –2.5 SDs, and –3 SDs). No significant difference was found between the two groups.

**Table 2:** Relative risk, with 95% CIs, of microcephaly by severity in offspring of women monoinfected with Zika virus and women infected with both Zika virus and chikungunya virus during gestation in the Microcephaly Epidemic Research Group Pregnant Women Cohort

	ZIKV		ZIKV + CHIKV			
	Total	*n* (%)	Total	*n* (%)	RR (95% CI)	*P*-Value
Microcephaly at first evaluation –2 SD	198	5 (2.53)	80	2 (2.50)	0.99 (0.19–4.99)	0.99
Microcephaly –2 SD at any time during follow-up	198	13 (6.57)	80	2 (2.50)	0.38 (0.09–1.65)	0.17

CHIKV = chikungunya virus; ZIKV = Zika virus.

In considering the timing of rash in relation to microcephaly detected at any time during follow-up, among the group infected with both ZIKV and CHIKV, the mothers of two children with microcephaly presented with a rash in the second and third trimesters of gestation. Among the mothers of the 13 children with microcephaly in the ZIKV-monoinfected group, one presented with a rash in the first trimester, seven presented with a rash in the second trimester, and five presented with a rash in the third trimester.

The median age at the neurological, ophthalmological, and audiological evaluation and dysphagia assessment was 13.3 months (P_25_–P_75_: 8.3–20.3 months); the median age at the imaging evaluation was 3.2 months (P_25_–P_75_: 2.3–5.3 months). There were no statistically significant differences between the monoinfected ZIKV group and the ZIKV/CHIKV group in the relative risks of congenital anomalies, such as microcephaly; neurological, ophthalmological, and audiological abnormalities; CNS imaging anomalies; or dysphagia, taken separately, considering a cutoff point of –2 SDs for microcephaly definition (Table 3). No difference was found between the ZIKV-monoinfected group and the ZIKV/CHIKV group for risk of premature birth, low birthweight, or small for gestational age status (Table 4).

**Table 3:** Relative risk, with 95% CIs, of congenital conditions in offspring of women monoinfected with Zika virus and women infected with both Zika virus and chikungunya virus during gestation in the Microcephaly Epidemic Research Group Pregnancy Cohort

	ZIKV		ZIKV + CHIKV			
Congenital manifestations	Total	*n* (%)	Total	*n* (%)	RR (95% CI)	*P*-Value
Neurological abnormalities						
At first evaluation	150	5 (3.33)	66	5 (7.58)	2.27 (0.68–7.58)	0.17
At any time	160	17 (11.33)	66	11 (6.67)	1.47 (0.72–2.96)	0.28
Ophthalmological abnormalities						
At first evaluation	198	8 (4.04)	80	4 (5.00)	1.08 (0.34–3.46)	0.89
At any time	198	70 (35.35)	80	21(26.26)	0.867 (0.28–2.65)	0.80
Audiological abnormalities						
At first evaluation	142	6 (4.23)	57	4 (7.02)	1.66 (0.48–5.66)	0.41
At any time	162	1 (0.62)	67	0	2.41 (0–94.2)	1.00
Central nervous system imaging anomalies						
At any time	112	6 (5.36)	52	5 (9.62)	1.79 (0.57–5.61)	0.31
Dysphagia						
At first evaluation	142	3 (2.11)	61	1 (1.64)	0.77 (0.082–7.32)	0.82
At any time	198	5 (2.53)	80	1 (1.25)	0.501 (0.59–4.21)	0.51

CHIKV = chikungunya virus; ZIKV = Zika virus.

Numbers varied because of missing values.

**Table 4:** Crude RR, with 95% CIs, of various perinatal outcomes in offspring of women monoinfected with Zika virus and women infected with both Zika virus and chikungunya virus during gestation in the Microcephaly Epidemic Research Group Pregnant Women Cohort

	ZIKV		ZIKV + CHIKV			
	Total	*n* (%)	Total	*n* (%)	RR (95% CI)	*P*-Value
Low birth weight (<2,500 g)	144	17 (11.81)	63	3 (4.76)	0.40 (0.12–1.32)	0.11
Low birth weight (≤2 SD)	144	8 (5.56)	63	1 (1.59)	0.28 (0.36–2.23)	0.21
Prematurity	169	35 (20.71)	74	9 (12.16)	0.58 (0.29–1.15)	0.11
Small for gestational age	144	20 (13.89)	63	5 (7.94)	0.57 (0.22–1.45)	0.23

CHIKV = chikungunya virus; CI = confidence interval; ZIKV = Zika virus.

The risk of abnormalities associated with congenital viral infection was investigated in three different ways. First, the number of children with at least one abnormality was determined. Sixteen of these were found in the monoinfected group (16/63), and 11 were found in the ZIKV/CHIKV group (11/36), indicating no significant difference (Table 5). The risk for the combined occurrence of two abnormalities was calculated, and no difference was found between the groups. The risk was not calculated for combinations of microcephaly and imaging abnormalities, microcephaly and ophthalmological abnormalities, or imaging and ophthalmological abnormalities, because these abnormalities were not found to co-occur. The risk of co-occurrence of three abnormalities was also calculated, and again, no significant difference was found between the two groups. The risk was not calculated for combinations of microcephaly, ophthalmological abnormalities, and neurological abnormalities; microcephaly, CNS imaging abnormalities, and ophthalmological abnormalities; or neurological abnormalities, ophthalmological abnormalities, and imaging abnormalities, because these combinations were not observed.

**Table 5:** Crude RR, with 95% CIs, of having at least one abnormality and various combinations of abnormalities in offspring of women monoinfected with Zika virus and women infected with both Zika virus and chikungunya virus during gestation in the Microcephaly Epidemic Research Group Pregnant Women Cohort

	ZIKV		ZIKV + CHIKV			
	Total	*n* (%)	Total	*n* (%)	RR (95% CI)	*P*-Value
Any of the outcomes*						
Microcephaly and/or central nervous system imaging abnormalities and/or neurological abnormalities and/or ophthalmological abnormalities	63	16 (25.40)	36	11 (30.56)	1.20 (0.62–2.30)	0.58
Two combined outcomes						
Microcephaly and neurological abnormalities	63	1 (1.59)	36	0	1.75 (0–68.25)^†^	1.00
Neurological abnormalities and CNS image abnormalities	63	1 (1.59)	36	0	1.75 (0–68.25)^†^	1.00
Neurological and ophthalmological abnormalities	63	1 (1.59)	36	1 (2.78)	1.38 (0.33–5.68)	0.68
Three combined outcomes						
Microcephaly and neurological and CNS image abnormalities	63	0	36	1 (2.78)	1.75 (0.044–+inf)^†^	0.36

CHIKV = chikungunya virus; CNS = central nervous system; CI = confidence interval; +inf = upper confidence interval limit not estimated; ZIKV = Zika virus.

* Total number of children evaluated for microcephaly, CNS imaging abnormalities, neurological abnormalities, and ophthalmological abnormalities.

^†^ The OR was calculated using a median unbiased estimator for binary data in an unconditional logistic regression model.

## DISCUSSION

In the present study, the risk of adverse effects was investigated in 278 children, 198 of whom were born to mothers monoinfected with ZIKV, and 80 of whom were exposed to ZIKV and CHIKV. No statistically significant differences were observed between the groups in the offspring’s risks of microcephaly, adverse birth outcomes (low birth weight, prematurity, small for gestational age), neuroimaging abnormalities, or other structural or functional alterations (dysphagia, neurological, ophthalmological, or audiological).

Some studies have revealed that maternal monoinfection by ZIKV and monoinfection by CHIKV may have severe neurological consequences for the newborn. However, there are still no studies in the literature on the effects of the infection by both viruses during the same gestation. Infants exposed to ZIKV infection during gestation may present a variety of congenital conditions ranging from asymptomatic infection to full microcephaly. In some cases, children who show no signs of microcephaly at birth may develop other abnormalities that can be observed in brain scans or detected during subsequent neurodevelopmental assessments.^13^ Driggers et al. (2016) described various clinical findings in the fetus caused by ZIKV infection and reported the presence of the virus in the brain, placenta, membranes, and umbilical cord.^16^ Other published studies have revealed a higher frequency of adverse events during gestation when maternal infection occurs during the first or second trimester. Honein et al. (2017) identified congenital anomalies in 11% of pregnant women with laboratory-confirmed ZIKV infection in the first trimester of gestation. In this cohort, there were no adverse outcomes when the infection occurred in later trimesters.^17^ In the case of CHIKV infection, Kury et al. (2022) presented two cases of infants born to mothers infected with CHIKV during the third trimester of gestation. Both infants tested IgM-positive for CHIKV. In the first case, the child presented with perioral cyanosis, crying, irritability, and signs of acute encephalitis in the magnetic resonance imaging (MRI) of the cranium. In the second case, the newborn presented with irritability, fever, and a maculopapular exanthem from the fourth day after birth. The disease progressed to necrotizing enterocolitis requiring surgery. Magnetic resonance imaging revealed evidence of acute encephalitis.^18^

In the present study, the manifestations found in children were more typical of congenital complications associated with ZIKV. In the group of monoinfected women, most cases of microcephaly (8/13) were identified in children whose mothers developed an exanthem during the first or second trimester of gestation. The mothers of the two children with microcephaly in the ZIKV/CHIKV group developed an exanthem in the second or third trimester of gestation. Neither child presented with characteristics compatible with manifestations of perinatal CHIKV infection. However, the small sample size prevents the authors from drawing conclusions about the role CHIKV may play in cases of gestational coinfection.

The authors of the present study considered microcephaly both as a separate outcome and in combination with other findings consistent with CZS. Outcomes were more likely to occur in isolation than in combination with other outcomes, both in the group of monoinfected women and in the ZIKV/CHIKV group, with no significant difference between the two groups. A meta-analysis of data from 13 cohorts of pregnant women with confirmed diagnosis of ZIKV infection during gestation from the Brazilian Zika Cohorts Consortium also revealed results that corroborate the finding that the risk of concomitant abnormalities is low. After various analyses, this meta-analysis revealed that only two of the 107 infants concomitantly presented with both neurological and ophthalmological abnormalities, and only four presented with both neurological and neuroimaging abnormalities.^13^

The study results indicate a lower risk of low birth weight, with a cutoff point of ≤2 SDs, in the group of children born to ZIKV/CHIKV-infected mothers than among those born to ZIKV-monoinfected mothers. The authors believe, however, that this difference can be attributed to the small sample size, as some studies have revealed that ZIKV infection may be associated with a high risk of low birth weight. In one case series of children exposed to ZIKV during gestation, the prevalence of low birth weight was found to be twice as high as that among babies not exposed to the virus.^19^ One study designed to produce an epidemiological characterization of ZIKV cases in Brazil in 2020 revealed that 44% of the infants included in the study presented with low birth weight, a finding consistent with CZS.^20^ Some studies conducted to investigate the effects of CHIKV in gestation revealed birth weights normal for infants born at term; none of these studies revealed any association between low birth weight and the infection under study.^21^^,^^22^ No studies were found in the literature in which the effects of coinfection on newborn weight at birth were investigated.

A comparison of the results for neurological abnormalities (at least one of irritability, altered muscle tone, dysphagia, and seizures) did not reveal any significant difference between the monoinfection and ZIKV/CHIKV groups. Fontes et al. (2025) compared the risk of adverse outcomes in children born to mothers infected with ZIKV during pregnancy with that of a group of children not exposed and found an OR of 6.95. These authors suggest that 84% of the neurological abnormalities encountered in the study may be associated with ZIKV infection during pregnancy.^23^ The most common neurological condition described in the literature in cases of vertical transmission of CHIKV is encephalitis.^24^ Bandeira et al. (2016) describe the case of a pregnant woman who, 4 days before delivery, presented with fever and skin rash. Her newborn developed a skin rash, fever, and hypoactivity 4 days after birth, progressing to generalized seizures. Reverse-transcription polymerase chain reaction test results on the cerebrospinal fluid, blood, urine, and saliva of the infant were positive for CHIKV and negative for ZIKV and DENV.^24^ Enter et al. (2018) describe three cases of infants infected with CHIKV by vertical transmission on the island of Curaçao. The first infant was born prematurely with an abnormal heart rate and died after cerebral hemorrhage. The serology result for CHIKV was positive. The second infant also tested positive for CHIKV and developed fever, skin rash, and seizures during the first days of life, with diffuse lesions revealed on an MRI scan of the CNS. The third infant presented with an abnormal heart rate and possible fetal distress during delivery, presenting with a skin rash and persistent irritability a few days after birth. This child’s serum also tested positive for CHIKV.^25^ No reports were found in the literature indicating that neurological manifestations are associated with ZIKV/CHIKV coinfection.

There was no significant difference between the children born to monoinfected pregnant women and those born to ZIKV/CHIKV-infected pregnant women in relation to ophthalmological abnormalities, although some such anomalies have been described in cases of CZS and have also been reported in individuals with postpartum CHIKV infection. One study of infants born to women infected with ZIKV during pregnancy revealed that one-third of the children with CZS presented with ophthalmological manifestations. Of 938 eyes examined, 269 exhibited chorioretinal scarring, focal pigmentary changes, and optic nerve pallor, these being the most frequent manifestations.^26^ Da Silva et al. (2022) conducted a systematic review and found that ophthalmological symptoms and manifestations may occur in cases of postnatal CHIKV infections. These authors found that the most common ophthalmological symptoms in individuals with CHIKV are pain, inflammation, and reduced visual acuity. The most common manifestations were conjunctivitis and optic neuritis.^27^ No reports of ophthalmological manifestations of CHIKV congenital infection were found.

The present study did not reveal any differences between the groups in relation to the audiological outcomes investigated. Neither was it possible to compare the study results with those of other studies, owing to the limited literature available.

No difference was observed between the groups studied regarding CNS anomalies. Brasil et al. (2016) reported several CNS imaging abnormalities, including brain calcifications, increased ventricular dilation, and hypoplasia of various brain structures, in children born to mothers exposed to ZIKV during pregnancy.^28^ Likewise, Fontes et al. (2025) found the likelihood of CNS imaging abnormalities to be almost 13-fold greater in children exposed to ZIKV during gestation, with calcifications identified only in the exposed group.^23^ One study of nine encephalopathic children born to mothers infected with CHIKV during pregnancy revealed nuclear MRI anomalies suggestive of cytotoxic and vasogenic edema in certain regions of the brain, as well as reperfusion in areas of previous hypoperfusion.^29^

One possible explanation for the absence of differences between the groups for most of the outcomes examined may be the timing of the infection during pregnancy. Some studies have indicated that children born to mothers infected with Zika during the first or second trimester have a greater frequency and severity of adverse outcomes than those born to mothers infected during the final stages of gestation.^13^^,^^28^ In the case of CHIKV, the consequences for the newborn appear to be more common and more severe when mothers are infected in the third trimester, the highest risk being associated with infections occurring closer to the time of delivery. In the present study, although 40% of rashes detected in ZIKV/CHIKV-infected women occurred during the third trimester, the predominant clinical manifestations were more consistent with ZIKV infection. The study data provide no evidence that infection by one virus exacerbates the effect of the other. There is, therefore, a need for further investigation of whether, in coinfection by two or more arboviruses, one virus predominates over the other or the two act in synergy to produce symptoms and other adverse outcomes, including vertical transmission.^30^

One limitation of the present study was the relatively small sample size. However, the study population consisted of a cohort of 503 pregnant women who presented with an exanthem put together during the ZIKV epidemic in Pernambuco with the aim of investigating adverse events during pregnancy. While data collection was underway, CHIKV began to circulate more intensely in the region, leading to the cases of coinfection or sequential infection included in the present study. This was the largest sample size possible; however, there may have been a problem related to power to detect the difference in risk for some variables. For example, the post hoc power calculation to detect a difference in the risk of microcephaly was 31% (β error of 69%), considering microcephaly at any time. Therefore, because the power was low, it cannot be ruled out that there is a difference in risk between the groups. Another potential limitation concerns the definition of exposure in the groups. For the monoinfection group, the criteria adopted were those proposed by Ximenes et al. (2019) and Ximenes et al. (2021), who ranked the degree of evidence of infection in pregnant women, in accordance with the methods used in studies published by the MERG and the WHO.^12^^,^^14^ Infection with CHIKV in the second coinfected group was determined using criteria similar to those used by Ximenes et al.^12^^,^^14^ On the basis of the type of examination available for each pregnant woman (qRT-PCR and IgM), a team of MERG specialists (D. de Barros Miranda-Filho, R. Arraes de Alencar Ximenes, U. Ramos Montarroyos) ranked various forms of evidence of infection. This established various levels of evidence of infection. It is important to highlight the difficulty in establishing whether these were true coinfections or sequential infections. There are several major challenges: 1) because there is a possibility of asymptomatic infection, the rash timing could be missed for one of the two infections; 2) it cannot be easily clinically distinguished whether the rash was caused by ZIKV or CHIKV; 3) only the maternal test results are available, so it is difficult to determine how long the fetus had a viremia; 4) there is limited evidence on the duration of viremia in fetuses. Some evidence indicates a long persistence, so it is plausible that some of these were true coinfections. In the present study, cases for which it was not possible to ascertain whether infection had occurred during pregnancy were excluded. The low frequency of adverse events in the study sample and a potential problem related to power to detect the differences between groups highlight the importance of creating consortia between research groups and sharing data from several studies to conduct individual participant data meta-analysis for Zika, as well as for other emerging diseases, such as the Zika Brazilian Cohorts Consortium and the WHO.^31^

## CONCLUSION

Although previous studies have revealed that both infections, when they occur independently, may be associated with adverse health outcomes in newborns, the present study did not, in general, reveal differences in the frequency or type of adverse effects among children born to mothers infected with both ZIKV and CHIKV during pregnancy. Given the limited amount of information in the literature on the consequences of ZIKV and CHIKV coinfection, the authors recommend using molecular arbovirus panels during surveillance of acute febrile illnesses in pregnancy to inform future research designed to answer this question. The present study provides valuable preliminary evidence that coinfection, or sequential infections, are a reality in settings with cocirculation of CHIKV and ZIKV. Therefore, expectant parents should be counseled on how to protect themselves from vector insect bites, considering the potential risks of permanent sequelae in affected children.
